# Author Correction: Novel peptide inhibitor of human tumor necrosis factor-α has antiarthritic activity

**DOI:** 10.1038/s41598-024-65261-4

**Published:** 2024-06-21

**Authors:** Debasis Sahu, Charu Gupta, Ragothaman M. Yennamalli, Shikha Sharma, Saugata Roy, Sadaf Hasan, Pawan Gupta, Vishnu Kumar Sharma, Sujit Kashyap, Santosh Kumar, Ved Prakash Dwivedi, Xiangli Zhao, Amulya Kumar Panda, Hasi Rani Das, Chuan-Ju Liu

**Affiliations:** 1 Product Development Cell, National Institute of Immunology, New Delhi, India; 2 Department of Orthopedics Surgery, New York University School of Medicine, New York, NY USA; 3 School of Biomedical Sciences, Galgotias University, Greater Noida, UP India; 4 Department of Bioinformatics, School of Chemical and Biotechnology, SASTRA Deemed to Be University, Thanjavur, Tamil Nadu India; 5 Amity Institute of Forensic Sciences, Amity University, Noida, Uttar Pradesh India; 6 CSIR-Institute of Genomics and Integrative Biology, Delhi, India; 7 Department of Pharmaceutical Chemistry, Shri Vile Parle Kelavani Mandal’s Institute of Pharmacy, Dhule, Maharashtra India; 8 Department of Pharmacoinformatics, National Institute of Pharmaceutical Education and Research (NIPER), Mohali, Punjab India; 9 Division of Pediatric Rheumatology, University of California San Francisco, San Francisco, CA USA; 10 Department of Genetics, University of Delhi, Delhi, India; 11 Immunobiology Group, International Centre for Genetic Engineering and Biotechnology (ICGEB), New Delhi, India; 12 Science Habitat, Ubioquitos Inc, London, ON Canada; 13 Department of Orthopaedics and Rehabilitation, Yale University School of Medicine, New Haven, CT USA

Correction to: *Scientific Reports* 10.1038/s41598-024-63790-6, published online 05 June 2024

The original version of this Article contained an error in Affiliations 3 and 13.

Affiliation 3:

‘Clinical Research Division, School of Basic and Applied Sciences, Galgotias University, Greater Noida, Uttar Pradesh, India.’

now reads,

‘School of Biomedical Sciences, Galgotias University, Greater Noida (UP), India.’

Affiliation 13:

‘Department of Orthopaedics, Yale School of Medicine, New Haven, CT, USA.’

now reads,

‘Department of Orthopaedics and Rehabilitation, Yale University School of Medicine, New Haven, CT, USA.’

The original Article has been corrected.

