# Supplementary material for: Novel peptide inhibitor of human tumor necrosis factor-α has antiarthritic activity
Source: Sci Rep. 2024 Jun 5;14:12935. doi: 10.1038/s41598-024-63790-6 (PMC11153517; doi:10.1038/s41598-024-63790-6)
Supplement: Supplementary file 1 — Supplementary Legends. [file 41598_2024_63790_MOESM1_ESM.docx]

**Supplementary figure legends**

**Figure S1.** The stretches of residues are present at the interface of TNF monomeric chains. The residues involved in the interaction are shown in colored and upper-case letters.

**Figure S2.** (A) Structure of the human anti-TNF-α peptide synthesized using solid-phase F-moc method. (B) The mass spectrometric profile of the purified peptide was analyzed using a MALDI TOF/TOF mass spectrometer. The molecular weight of the peptide is 993.45, as indicated. (C) Reverse-phase-HPLC profile of the anti-TNFα peptide after purification. The peak indicates the retention time.

**Fig S3.** Inhibition of cell surface binding of TNF on U937 (human monocyte cell line) cells. The anti-TNFα peptide inhibits TNF-α binding to U937 human monocyte cells. The green signal is due to the TNFα binding to the membrane receptors on U937 cells (A). Cells not stimulated with TNFα, (B) Cells incubated with TNFα (100 ng/ml), and (C) Cells treated with the premixed TNFα and anti-TNFα peptide decrease the green signal (C).

**Figure S4.** Histogram from FACS enrichment analysis showing the fluorescence intensities of (A) cells not stimulated with LPS, (B) cells stimulated with LPS (1 μg/ml), and (C) LPS (1 μg/ml)-stimulated cells followed by treatment with the peptide (200 μM) causing inhibition of TNF expression/binding on the cell surface.

**Figure S5.** Western blot of nuclear extract after TNFα induced NFκB translocation in A549 cells and its inhibition by the peptide at 50, 100, and 200 μM. Uncropped blot membrane images of (A) NFκB and (B) β-actin blots. Fig S5C and D show the complete pictures of NFκB and β-actin blot membranes as observed in the imager.
