# Supplementary material for: Novel peptide inhibitor of human tumor necrosis factor-α has antiarthritic activity
Source: Sci Rep. 2024 Jun 5;14:12935. doi: 10.1038/s41598-024-63790-6 (PMC11153517; doi:10.1038/s41598-024-63790-6)
Supplement: Supplementary file 2 — Supplementary Figure S1. [file 41598_2024_63790_MOESM2_ESM.pdf]

Figure S1.

Interaction between A and B chains.

Chain A:

`57LiysqvlfkgqgcpsTHvLlthtisRiavsYqtkvNLLSaIKSpcQRetpegaeakpwyepiYLGGVFQlekgdr`
`lsaeinrpdyldfaesgqvyfgiiaL 157`

Chain B:

`11KpvaHvvanpqaegqlqwlnrrANaLlangvelrdnqlvvpseglyLiYsQvLfkgqgcpsthvllthtisriav`
`syqtkvnllsaiKspcqrEtPegAeaKPWYePiYlggvfqlekgdrlsaeinrpdyldfaeSGQvYfgiIaL 157`

Interaction between A and C chains.

Chain A :

`6RTPsdkpVaHvvanpqaegqlqwlnrraNallangvelrdnqlvvpseglyliYsQvlfkgqgcpsthvllthtis`
`riavsyqtkvnllsaikspcqrEtpegaeaKPwYePiYlggvfqlekgdrlsaeinrpdylDfaEsGqvYfgiIaL`
`157`

Chain C:

`54GLyliysqvlfkgqgcpstHvLlthtisriavsyqtkvnLLSAIKSpCQRetpegaeakpwyepiYLGgVFQlek`
`gdrlsaeinrpdyldfaesgqvyfgiiaL 157`

Interaction between B and C chains.

Chain B:

`55LyliysqvlfkgqgcpstHvLlthtisriavsyqtkvNLLSaIKSpcQRetpegaeakpwyepiYLGGVFQlekg`
`drlsaeinrpdyldfaesgqvyfgiiaL 157`

Chain C:

`6RtPSdkpVaHvvanpqaegqlqwlnrraNaLlangvelrdnqlvvpseglyliYsQvlfkgqgcpsthvllthtis`
`riavsyqtkvnllsaiKspcqrEtpegaeaKPWYePiYlggvfqlekgdrlsaeinrpdyldfaeSGQvYfgiIaL`
`157`
