# Supplementary material for: Novel peptide inhibitor of human tumor necrosis factor-α has antiarthritic activity
Source: Sci Rep. 2024 Jun 5;14:12935. doi: 10.1038/s41598-024-63790-6 (PMC11153517; doi:10.1038/s41598-024-63790-6)
Supplement: Supplementary file 3 — Supplementary Figure S2. [file 41598_2024_63790_MOESM3_ESM.pdf]

Figure S2.

A

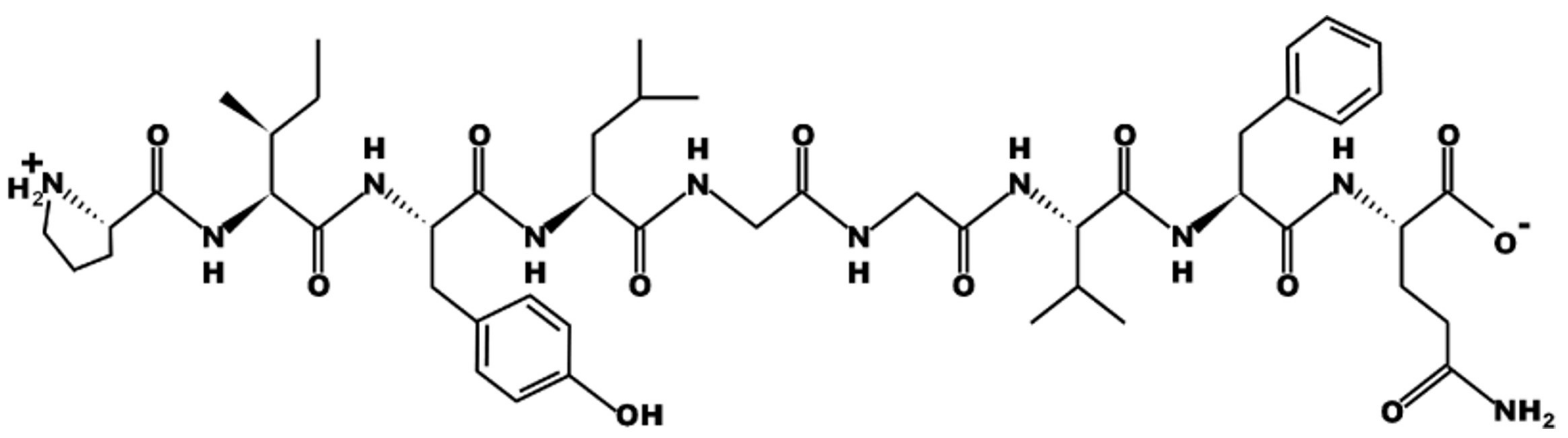

B

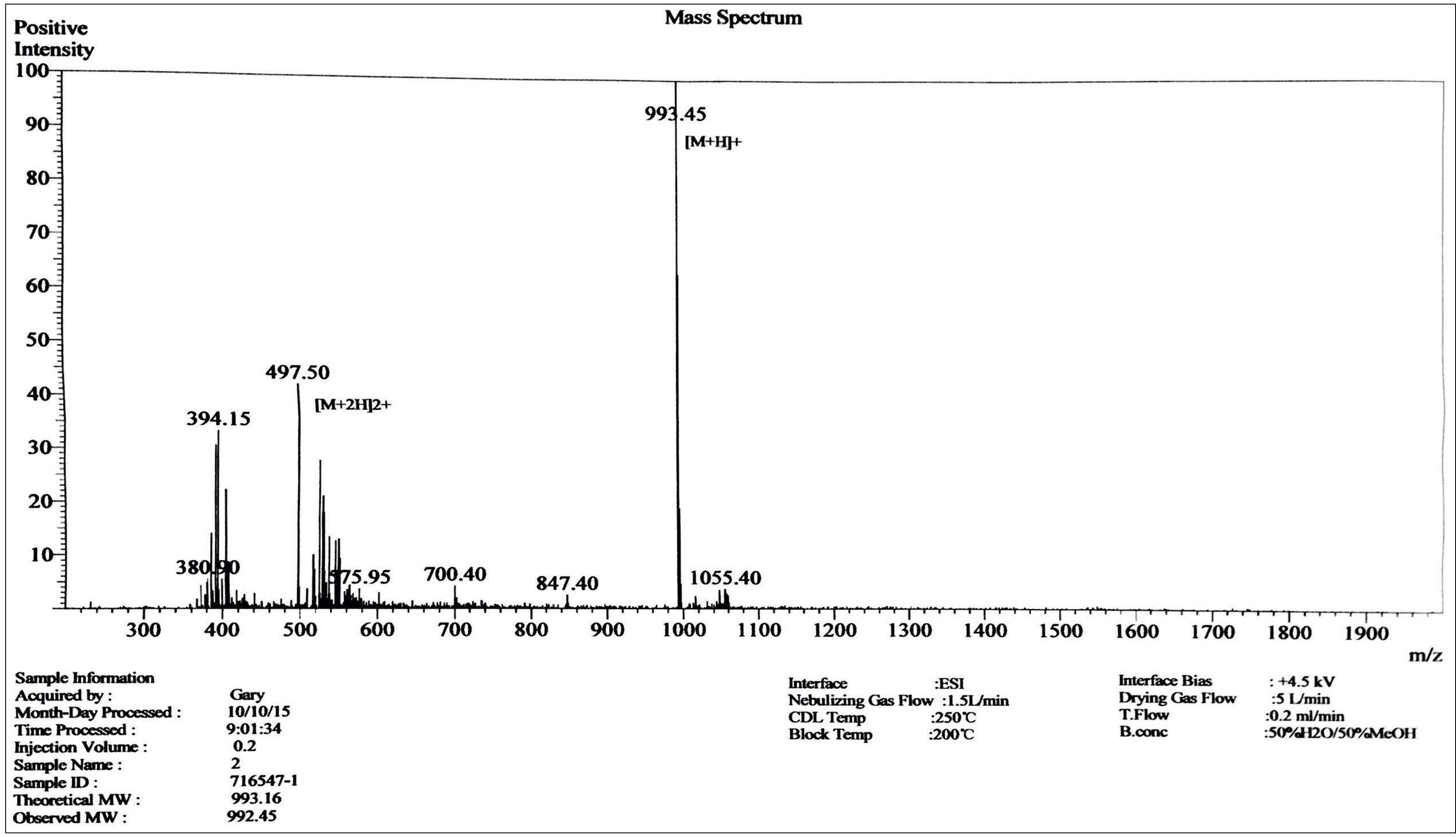

C

Pump A : 0.065% trifluoroacetic in 100% water (v/v)
Pump B : 0.05% trifluoroacetic in 100% acetonitrile (v/v)
Total Flow:1 ml/min
Wavelength:220 nm

Time	Unit	Command	Value	Comment
0.01	Pumps	Pump A B.Conc	5	
25.00	Pumps	Pump A B.Conc	65	
25.01	Pumps	Pump A B.Conc	95	
31.00	Pumps	Pump A B.Conc	95	
31.01	Pumps	Pump A B.Conc	5	
40.00	Pumps	Pump A B.Conc	5	
40.01	Controller	Stop		

<<Column Performance>>
<Detector A>
Column : AlltimaTM C18 4.6 x 250 mm

Chromatogram

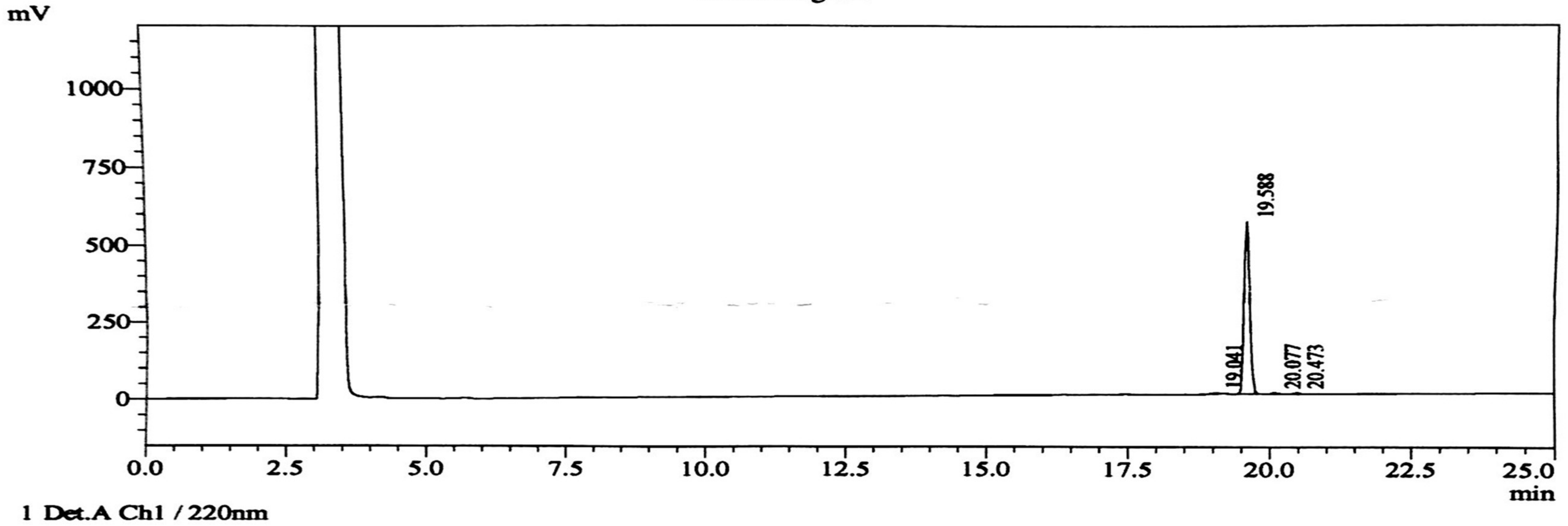

Peak Table

Detector A Ch1 220nm

Peak#	Ret. Time	Area	Height	Area %
1	19.041	28680	3698	0.726
2	19.588	3888680	555298	98.395
3	20.077	16322	3069	0.413
4	20.473	18409	3338	0.466
Total		3952092	565403	100.000
