# Supplementary figures and images for: Novel peptide inhibitor of human tumor necrosis factor-α has antiarthritic activity
Source: Sci Rep. 2024 Jun 5;14:12935. doi: 10.1038/s41598-024-63790-6 (PMC11153517; doi:10.1038/s41598-024-63790-6)

**Figure S3**

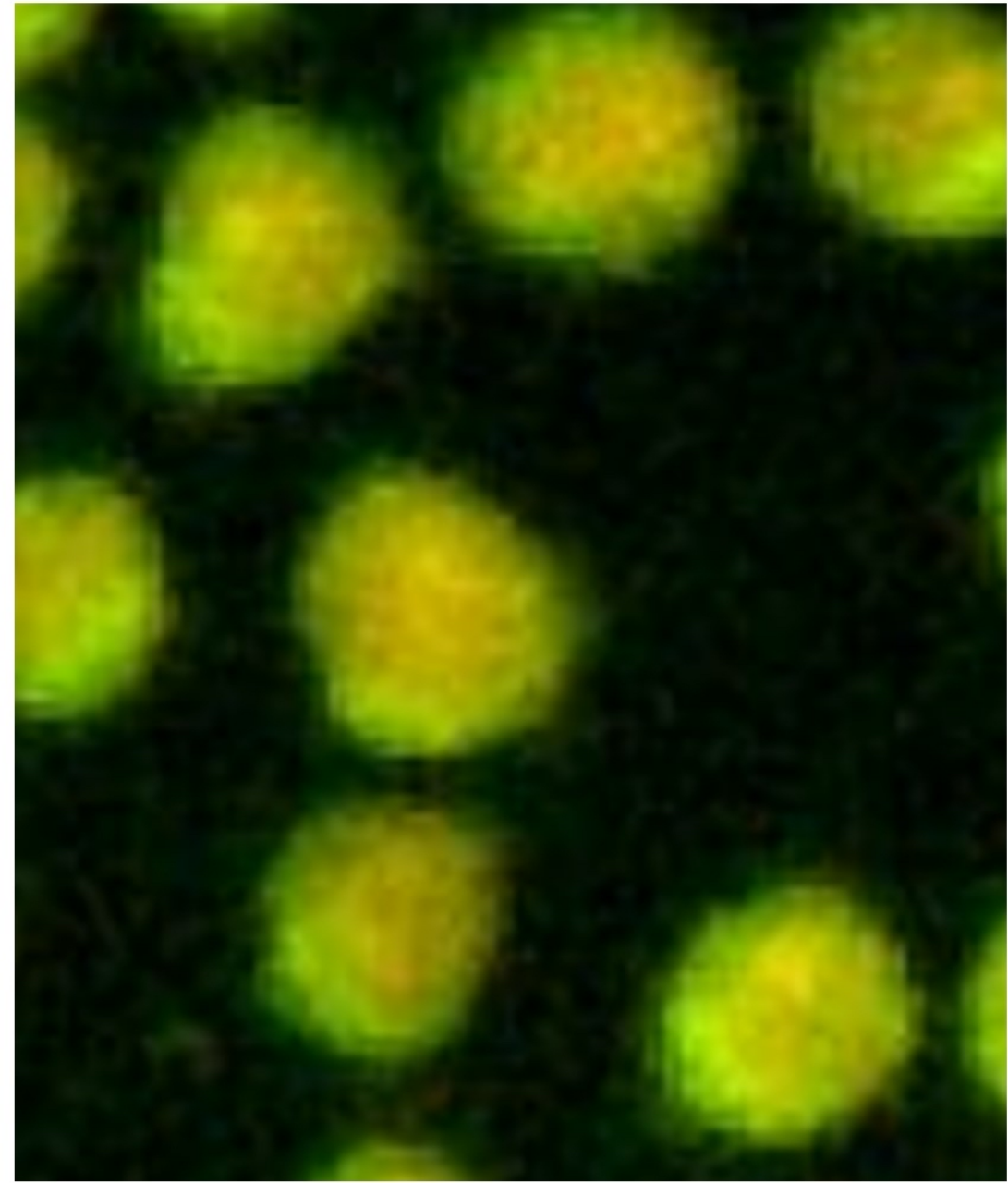

**Unstimulated**

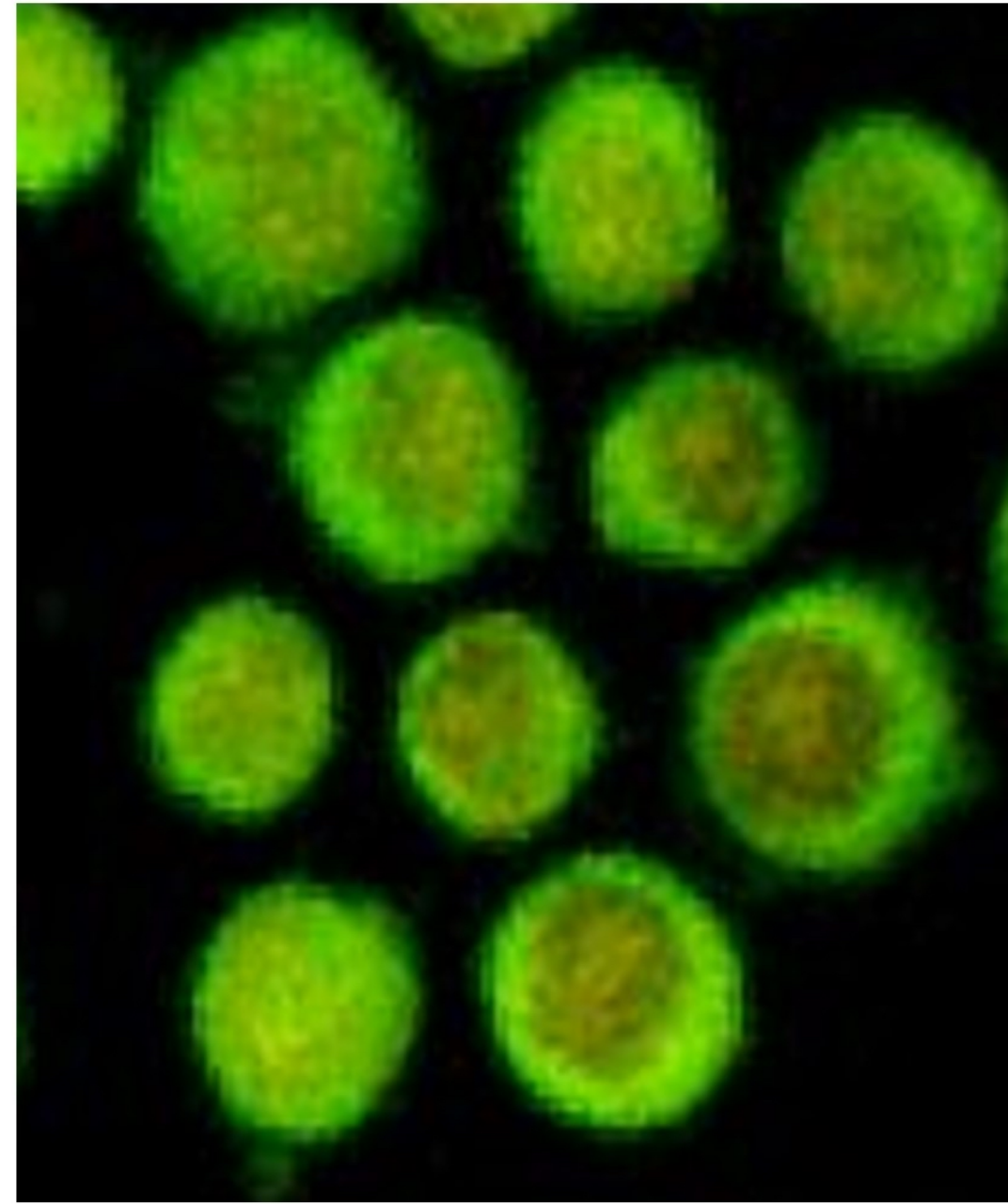

**TNF (100ng/ml)**

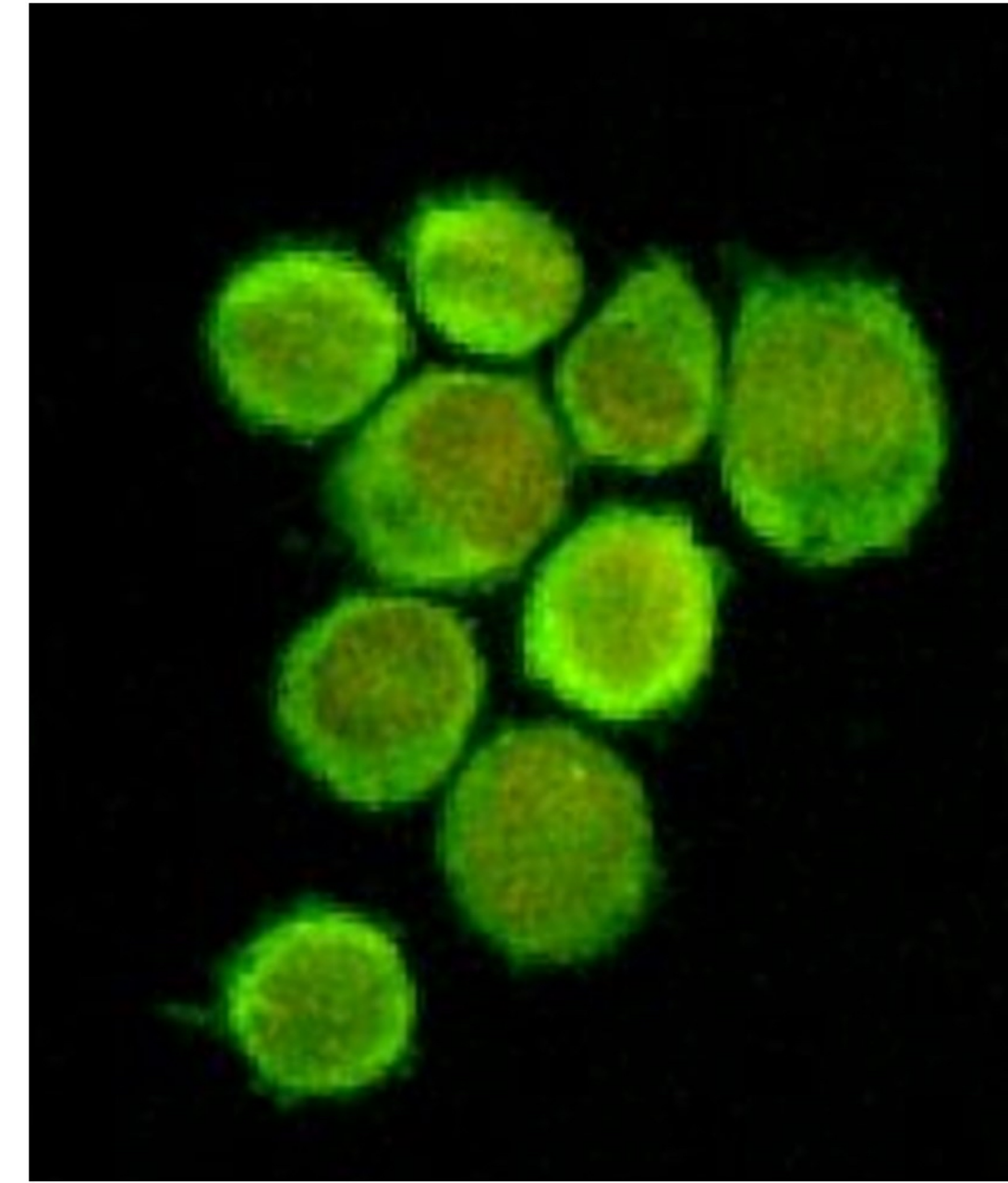

**TNF (100ng/ml)+  
Pep (100μM)**

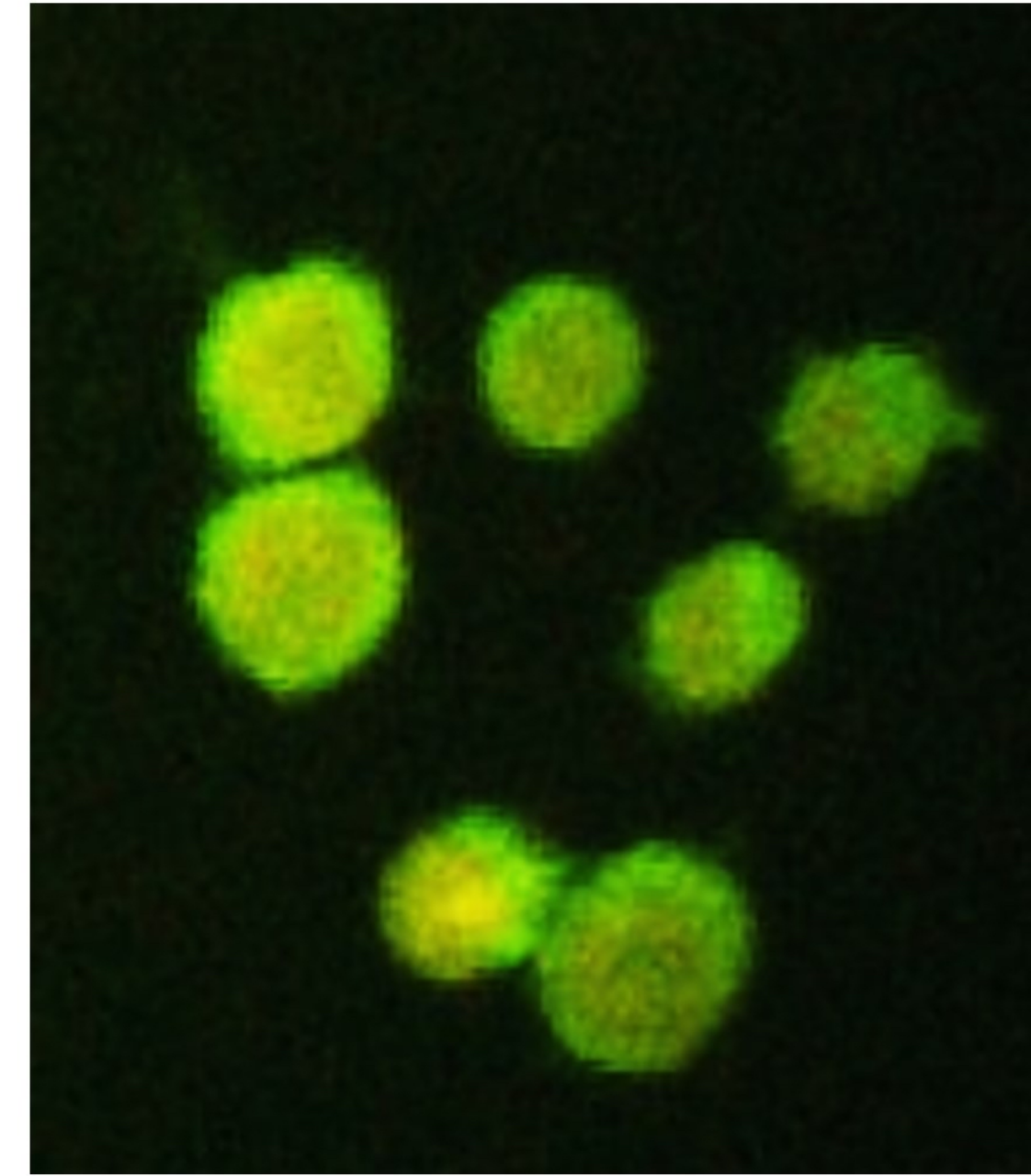

**TNF (100ng/ml)+  
Pep (200μM)**

Supplement: Supplementary file 4 — Supplementary Figure S3. [file 41598_2024_63790_MOESM4_ESM.pdf]

**Figure S4.**

**A. Unstimulated**

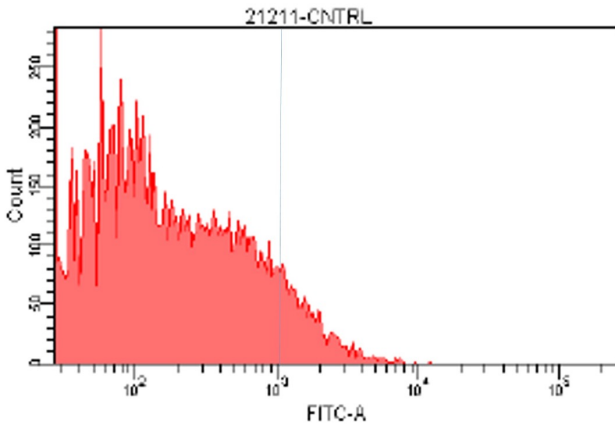

**B. LPS (1 $\mu$ g/ml)**

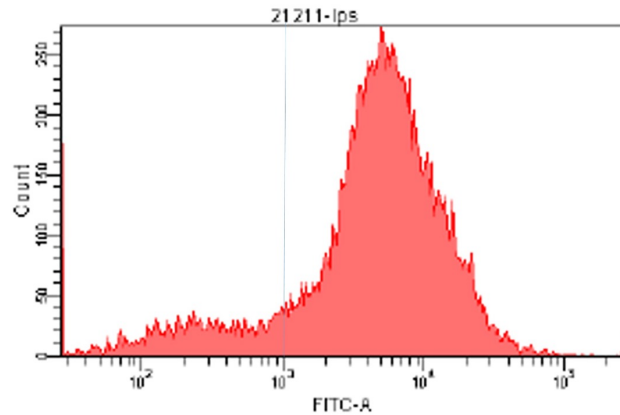

**C. LPS (1 $\mu$ g/ml) + Peptide (200  $\mu$ M)**

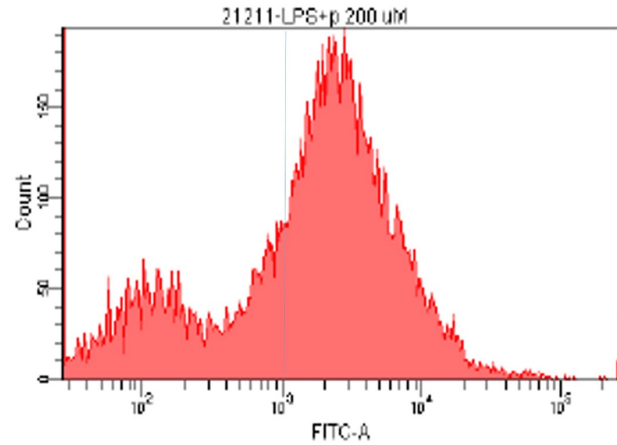

Supplement: Supplementary file 5 — Supplementary Figure S4. [file 41598_2024_63790_MOESM5_ESM.pdf]

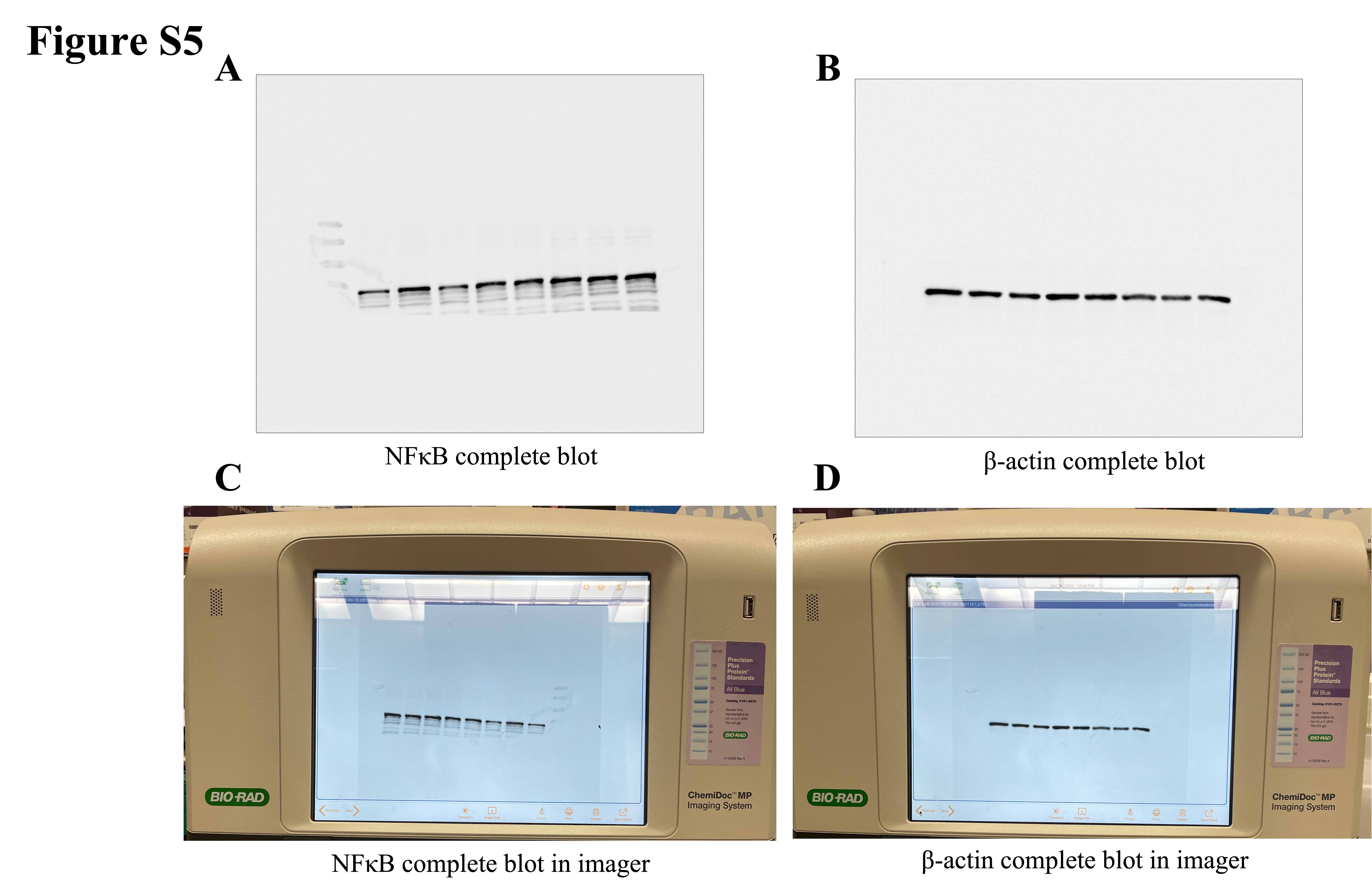

Supplement: Supplementary file 6 — Supplementary Figure S5. [file 41598_2024_63790_MOESM6_ESM.jpg]
